# Anæsthesia and Anæsthetics

**Published:** 1860-11

**Authors:** Edward R. Squibb

**Affiliations:** Brooklyn, N. Y.


*Anæsthesia and Anæsthetics.* By EDWARD R. SQUIBB, M. D., of Brooklyn, N. Y.

The condition of insensibility to pain belongs exclusively to the brain proper, or to that part of the nervous system which provides for sensation and voluntary motion; and is effected when not the result of mechanical injury, invariably through the agency of the circulation. It therefore follows upon this, and upon the circumstances that the nervous centres of organic life exercise no primary function of ordinary sensation or voluntary motion, that the special agents resorted to for anæsthetic purposes should not only be directed especially to the sensorium, but should be diverted as far as practicable from the remaining portions of the nervous system, in effect. But, the circulation carries the anæsthetic agent everywhere, and with the elements of vitality and molecular reproduction must convey and distribute this powerful agency also; and hence the special agent for effecting anæsthesia should not only act directly, promptly, and transiently upon the sensorium, but should be, as far as possible, at least, innoxious elsewhere. In short, it should suspend the functions of the sensorium without liability to interference with any other organ or function.

Such an anæsthetic effect is produced perhaps in the greatest degree of perfection by a certain amount of concussion of the brain, which sometimes results from accidental violence; and the effect is most perfect here, because it is produced directly upon the brain without any contamination of the circulation with foreign influences; and the circulation thus left free for the performance of its normal functions not only preserves the organic life intact during the temporary abstraction of the presiding sensorial functions, but through its reparative agency quickly remedies the shock, and restores the brain to its normal condition.

The next most perfect anæsthetic effect is that to which a small proportion of persons are susceptible, wherein the sensibility to ordinary impressions of pain or injury is suspended or overpowered through concentric nervous effect. Whenever the balance of nervous power is so disturbed as to reverse the current of the nervous batteries (so to speak), as in the so-called mesmeric condition of certain persons of feeble nervous tone or energy; and in the high degree of nervous excitement to which others are liable through agencies that act altogether from without, the æsthetic functions of the sensorium, are altogether suspended, as in catalepsy, or are so impaired that serious injuries are unconsciously received.

The effect, however, in both these classes of cases can never be utilized if from no other cause than because it is independent of the circulation, and all other practical means of production, maintenance, and control. The circulation therefore becomes indispensable as the means of introducing the anæsthetic agent, and of controlling its effects; and the collateral circumstance that the circulation must inevitably carry the agent to parts where it is not desired, and where it may become noxious, must be taken as a drawback, and a most important indication in both the selection and management of the anæsthetic to be used.

From these circumstances, and inductions taken as points of departure, it is not difficult to deduce the indications in the use of anæsthetics as being, first to suspend sensation and voluntary motion; and, secondly, to do this with the least possible interference with the functions of organic life. These points admitted, and kept prominently in view, will, with a little reasoning, render the management of anæsthetic agents very simple, and will make the accidents and mismanagements more intelligible and more easily avoided.

These accidents are, first in importance as well as in frequency perhaps, some form or degree of asphyxia. All the vapors used for anæsthetic purposes are irrespirable. That is, they do not contain oxygen in a condition in which it is available in the lungs for the renewal of the blood. Just in proportion, therefore, as the vapor is introduced is the normal quantity of air diminished, and the proper oxydation of the blood prevented; and the ratio of this proportion is as inevitable in the effect upon the powers of life as it would be if carbonic acid or water, or any other irrespirable medium was substituted even up to that proportion which produces spasmodic closure of the glottis. It has been not unfrequently noticed, in what the writer believes to be the mismanagement of both the common anæsthetics, that the administration has commenced with a proportion of the vapor so large as to produce this spasmodic closure of the glottis. Under such circumstances, if it was possible to keep up such a proportion throughout the struggling of the patient, the spasmodic closure would doubtless be as persistent as it is drowning. But when from withdrawing the sponge a little, or from the displacement of it in struggling, the proportion of air is increased, the glottis is relaxed again, and the imperfect respiration goes on quickly to a point when, from the undue, sudden and depressing effect of the anæsthetic on the nervous centres, the glottis no longer responds to the action of the irritant, and the vapor passes freely into the lungs, no matter how strong or how small the proportion of air mixed with it. The pulse and respiration then give the indications to suspend and reapply the anæsthetic, and it becomes a matter of time, endurance and of management as to how far the powers of life are taxed.

It is, therefore, not a question as to whether aeration of the blood is to be interfered with at all, or not, since some portion of anæsthetic vapor is indispensable, and since that portion must exclude a corresponding portion of the air; but the question is rather, how far the due aeration of the blood may be judiciously and safely interfered with; or in other words, what degree of asphyxia is justifiable and proper in the management of anæsthetics; and the natural conclusion is as practical as it is logical, namely, that the least possible degree is safest and best, and that the interference should not be hurriedly induced, or maintained a moment longer than is absolutely necessary.

If suspended animation from the circulation of venous blood in the brain was to be resorted to for anæsthesia, it would be necessary to immerse the patient at intervals in water, carbonic acid, or other irrespirable medium, in order to maintain the condition; and the risk of fatal asphyxia would be here much more apparent, though really not very much more imminent than in the nearly parallel case wherein the irrespirable vapor of ether is substituted throughout a clinical lecture, with the antagonistic stimulant effect of the operation postponed till near the end of a long period of insensibility. The position that the insensibility in ordinary anæsthesia is due to the circulation of unrenewed blood in the brain is, however, only true in part at the utmost, and this introduces another of the accidents that may occur in the management of anæsthetics.

If it were possible to separate the true desirable anæsthetic effect from every vestige of asphyxia on the one hand, and from ell direct interference with the functions of organic life on the other, it would probably be found to consist in a simple specific paralysis of the nervous ganglia of sensation ; and the desirable degree of such effect would be that which did not at alloverreach the object. By overreaching the object however, whether it be by a too profuse, or a too prolonged use of the agent, the result must be injurious, since suspended function is but one step in the catenation which leads to organization and death, and that step once passed, the others may be accomplished insidiously. Such an hypothetical position is, however, only assumed to show that there must be a condition of hyperanæsthesia, or excessive anæsthetic effect—that such a condition is hurtful and unsafe, and that it should be avoided by skill in management, no matter how safe the agent used may be considered.

That such conditions do not occur without washing through the failing functions of organic life, is the fortunaete result of the harmony and dependent action of the nervous centres, and these functions of organic life are commonly and very properly watched, as the means of control in the administration of anæsthetics. But apart from the fact that a most hurtful and dangerous degree of asphyxia may be induced suddenly, and while both pulse and respiration are spasmodically kept up by the stimulus of the first effect of the agent used, there are grave accidents which occur to the centres of organic life, both by reflex action from the brain proper, and by the presence in the circulation of such powerful depressing agents. Blood overcharged with anæsthetic vapors, and particularly when imperfectly aerated and slowly circulated, must necessarily fail of its due impression upon the cardice and respiratory ganglia, and paralysis of the heart, or muscles of respiration are, therefore, the common fatal accidents of anæsthetic practice.

All these circumstances lead directly to the conclusions, first, that anæstics should be given slowly and carefully, with free, unlimited admixture of air, so that there should never be any choking or spasmodic action of the glottis.—Secondly, that they should only be given at the time when the effect is needed, and be abandoned the moment the necessity is passed. Thirdly, that not only should the pulse and respiration be watched carefully during the whole period of insensibility, and be kept as near the normal standard as possible, but the slightest amont of blueness or lividity should be regarded as an indication of asphyxia, and be promptly responded to by a more free admission of air.

In the choice between the two anæsthetics in common use, one or two points are deserving of attention.

Chloroform is much less liable to produce cyanosis or asphyxia, because it is effective in much smaller quantity than ether, and does not therefore displace so much air in the respiratory process. The writer has never noticed any degree of blueness from the use of chloroform, but has often seen it in the use of ether. On the other hand, unless chlorofform be given with far more care than is necessary with either, it is, from its greater efficiency, much more liable to produce hyperanæsthsia, and to paralyse the heart and respiratory musbles. Hence chloroform, under ordinary circumstances, must be considered more dangerous to life, because its greater efficiency and activity, while they render it less liable to produce asphyxia, render it more liable to produce the other accidents of anæsthetic practice. The balance against it is, however, more applicable to its common and indiscriminate use than when applied with the care and precaution indicated in the foregoing remarks; and there is probably quite a large class of cases in which it cannot judiciously be replaced by any other agent, as, for instance, in parturition; in uremic convulsions of gestation and parturition; and, in short whenever an intermittent and prompt effect are desirable, and the due precautions can be rigidly observed. In careful practice, with ordinary good judgment and observation, it has, in the writer's opinion, the advantage over ether in every point except the single important one that, in rare instances, it is liable to produce sudden fatal paralysis of the heart.

Ether has been regarded as so safe an anæsthetic, that it is scarcely admitted as susceptible of doing harm; and the impression is very common that it can never endanger life.—That either of these propositions can be accepted admits of great doubt.

In the asphxia from drowning, if the immersion be of short duration, and if the muscular system has not lost its vital tonicity, it is usually only necessary to re-establish the respiration and circulation for a short time, by artificial means, to restore life. If that drowning be prolonged, however, by repeated short immersions, so that the same inefficient condition of the circulating blood be brought about during a half or three-quarters of an hour of struggling, and with depressing influences from other sources, as of previous disease or injury, so that the powers of endurance are worn out, and passive exudations are permitted to accumulate and obstruct the pulmonory air cells, the result would probably be very different. A condition of vital depression would be established which might very slowly go either way in the balance between life and death, but which would probably, in case of other coinciding influences, as after a serious surgical operation, ultimately terminate fatally. The partial asphyxia produced by a prolonged etherization is a nearly parallel case under ordinary circumstances; and here, as in other instances, pernicious influences may be masked by the complication and remoteness of the results.

So strongly has the writer's attention been drawn to these circumstances, by seeing and hearing of the profuse and wasteful use of ether, that it is a prominent object of this article to invite the profession to a closer scrutiny and observation of the effects; and if two or three fluid ounces of ether be found to produce a safer and better effect than double that quantity, an important point will have been attained.

The method of administering ether adopted by some close observers, is one which appears well adapted to ensure a due admixture of air. A folded napkin is rolled into the form of a cylinder, or truncated cone, and secured at the overlapping edges by two pins. The larger end is made wide enough to cover the nose and mouth, the nose fitting into a notch, where the two edges of the napkin at the widest end fail to overlap. The opening at the small end should be at least one and a half inches in diameter, and the larger the better. If anything be needed to give stiffness and form to this cone, a piece of pasteboard laid between the folds of the napkin before it is rolled up, will accomplish this purpose. This cone is held in the hand of the person who gives the ether, and, as a matter of economy, it may be removed from the face during each expiration. About two fluid drachms of ether is poured upon the inside of the napkin at a time, and renewed as often as may be requisite.

In the administration of ether for anæsthetic purposes, at least three well marked stages are commonly observable, and the duration of each varies very much with the temperament of the individual, the condition of the stomach, and the quality of the ether used. One of these stages, namely that of excitement and delirium, and the only troublesome one, has been hitherto supposed to be shorter in proportion as the ether contained less alcohol; but some very recent observations made by the intelligent House Surgeon of the New York Hospital, Dr. Weir—though as yet very limited in number—would appear to indicate that the point of maximum, or best effect in this respect, may be overreached, or that a determinate small proportion of alcohol in the ether may be useful. At least, Dr. Weir has been very naturally led to this inference by the effect of giving a small amount of brandy before the anæsthetic; and subsequently by the use of alcoholic ether. He, however, states distinctly, that as yet his observations are not sufficiently numerous to be relied upon. An occasional accident in the prolonged use of ether, for the mention of which the writer is also indebted to Dr. Wier, is the occasional occurrence of a smart, ephemeral, irritative fever, which folllows within twenty-four hours. In view of the circumstance that the local effect of ether is irritant to the extent of producing vesication when confined upon delicate surfaces, it may be easily understood that its application over the large and delicate mucous lining of the bronchial ramifications, throughout an unusually tedious and difficult operation, might produce a transient inflamatory effect. In conclusion, the writer is aware that the character and drift of these remarks are directly at variance with the teachings of some very high authorities who regard the anæsrhetic condition as one of dead drunkenness, and who advise the rapid and copious administration of the ether, in order to reach this condition in the shortest possible time.

It appears singular to the writer, that those who are in the daily habit of making the most delicate distinctions in diagnosis, should fail to discriminate between the effects of poisonous doses of alcohol and the desirable condition in ordinary anæsthesia, since they certainly do not much more nearly resemble each other than the coma of narcotism resembles natural sleep.—*Southern Medical & Surgical Journal.*

